# Two-stage correction of type IV total anomalous pulmonary venous connection

**DOI:** 10.1186/s13019-017-0617-1

**Published:** 2017-07-06

**Authors:** Hunbo Shim, Ji-Hyuk Yang, Tae-Gook Jun

**Affiliations:** Department of Thoracic and Cardiovascular Surgery, Samsung Medical Center, Sungkyunkwan University School of Medicine, 81 Irwon-ro, Gangnam-gu, Seoul, 06351 South Korea

**Keywords:** Pulmonary vein, Type IV TAPVC, Stage operation

## Abstract

**Background:**

The small size of the pulmonary veins in infants increases the risk of pulmonary vein obstruction (PVO) after surgical repair of type IV total anomalous pulmonary venous connection (TAPVC). Here, we described the outcomes of our strategy, which delayed total correction after initial partial correction.

**Methods:**

We reviewed the data of patients who underwent total correction for type IV TAPVC. In total, 11 out of 103 patients with TAPVC had type IV TAPVC with biventricular physiology. Of these 11 patients, we retrospectively reviewed the data of 7 patients who underwent two-stage correction. Major pulmonary venous confluent chambers, with the exception of the left superior pulmonary vein (LSPV), were initially anastomosed to the left atrium (LA), followed by anastomosis between the LSPV and the LA auricle.

**Results:**

The median weight, age, and LSPV size were 4.3 kg (range, 3.5–5.4 kg), 40 days (range, 20–103 days), and 4.5 mm (range, 3.0–5.4 mm), respectively, during the first operation and 12.2 kg (range, 8.5–31.5 kg), 1,165 days (range, 280–3,250 days), and 9.8 mm (range, 8.0–12.3 mm), respectively, during the second operation. The median Qp/Qs was 1.61 (range, 1.22–1.65) and the median cardiothoracic ratio was 0.52 (range, 0.49–0.57) at second operation. The median interval between the operations was 1,094 days (range, 196–3,226 days). The median follow-up period was 22 month (range, 7–59 month). No mortality or major morbidities occurred after either operation. The median V_max_ at the LSPV anastomosis site was 1.0 m/s (range, 0.8–1.3 m/s) on predischarge echocardiography. This patency was maintained at the last follow-up, showing an identical median V_max_ of 1.0 m/s (range, 0.8–1.3 m/s). All 7 patients who underwent two-stage correction were in good condition, without any clinical symptoms of PVO.

**Conclusions:**

Our results suggest that leaving the isolated LSPV uncorrected during infancy and performing a second operation when the LSPV has grown adequately is a viable treatment option for patients with type IV TAPVC.

## Background

Total anomalous pulmonary venous connection (TAPVC) is a cyanotic defect in which all pulmonary veins drain into the systemic venous circulation. This is a rare disease, with an incidence of 1/10,000 live births, accounting for 0.7–1.5% of all congenital heart defects [1, 2]. Among all types of TAPVCs, type IV is especially rare, with an incidence rate of 5–13% [3–8]. This makes evaluating the effectiveness of treatment strategies challenging. Total correction of type IV TAPVC shows a high mortality rate [9–11]. Few studies have reported fair results of correction of major pulmonary venous drainage without correction of minor abnormal pulmonary venous drainage [4, 12]. One approach could be to leave small isolated pulmonary venous drainage uncorrected, considering that the small size of the pulmonary vein at the first operation could lead to obstruction after anastomosis [7, 13]. However, as this uncorrected isolated abnormal pulmonary drainage causes a left to right shunt, this uncorrected abnormal drainage should be corrected in some manner. This retrospective study describes our experience with a two-stage correction of type IV TAPVC, in which we performed 1) anastomosis of major drainage from the pulmonary venous confluent chamber to the left atrium (LA) and 2) anastomosis of the uncorrected isolated pulmonary vein to the LA auricle after the uncorrected isolated pulmonary vein grew adequately.

## Methods

### Study population

From February 1995 to July 2016, 103 patients who had TAPVC with biventricular physiology underwent repair surgery. Among these 103 patients, there were 11 patients with type IV TAPVC. In this report, we describe the anatomy of the pulmonary veins based on a system suggested by Chowdhury et al. [14], which was validated in a large series by St. Louis et al. [15]. Type IV A was characterized by a separate anomalous connection of veins from each lung: “2 + 2” pattern. Type IV B was characterized by isolated drainage of a solitary pulmonary vein, with the remaining three veins draining to a common site: “3 + 1” pattern. Of 11 patients with type IV TAPVC, 3 patients were excluded, as they underwent one-stage correction; 2 patients with type IV B (“3 + 1” pattern) underwent one-stage correction owing to the institutional strategy prior to 2002, and 1 patient with type IV A (“2 + 2” pattern) underwent one-stage correction owing to an evenly separated anomalous connection of the pulmonary veins from each lung, with bilateral and symmetrical connection. Out of 8 patients with type IV B (“3 + 1” pattern) for whom two-stage correction was selected, the data of 7 patients (4 male and 3 female) who underwent complete correction of all anomalous connections of pulmonary veins were reviewed retrospectively; 1 patient was still awaiting the second operation at the time of writing this manuscript.

### Subtype variants

In all 7 patients with type IV B (“3 + 1” pattern), only the left superior pulmonary vein (LSPV) separately drained into an innominate vein via a vertical vein. The rest drained into the right atrium, converging to a common chamber. In 5 patients, the common chamber was connected to the coronary sinus, and in 2 patients, the common chamber drained into the inferior vena cava through a vertical vein (Table 1).

**Table 1:** Preoperative data at each operations

Patients	Drainage site	Sex	1^st^ operation			2^nd^ operation						TI (days)
			Age (days)	BW (kg)	LSPV (mm)	Age (days)	BW (kg)	LSPV (mm)	Qp/Qs	sPAP/sSAP	CT ratio	
1	RPVs and LIPV to CS, LSPV to innominate vein	F	71	4.3	5.4	1,165	12.2	10	1.60	0.28	0.53	1,094
2	RPVs and LIPV to CS, LSPV to innominate vein	M	84	5.0	3.8	280	8.5	8.5	-	-	0.52	196
3	RPVs and LIPV to IVC, LSPV to innominate vein	M	20	3.5	4.4	647	11.0	9.6	1.22	0.28	0.53	627
4	RPVs and LIPV to CS, LSPV to innominate vein	M	103	5.4	3.0	401	9.8	8.0	1.65	0.41	0.57	298
5	RPVs and LIPV to CS, LSPV to innominate vein	F	26	4.2	4.9	1,852	17.5	9.8	-	-	0.51	1,826
6	RPVs and LIPV to CS, LSPV to innominate vein	M	40	5.0	4.5	1,471	19.4	12.3	1.62	0.24	0.49	1,431
7	RPVs and LIPV to IVC, LSPV to innominate vein	F	24	3.5	3.4	3,250	31.5	11.5	-	-	0.49	3,226
Median			40	4.3	4.4	1,165	12.2	9.8	1.61	0.28	0.52	1,094

*BW* body weight, *CS* coronary sinus, *CT ratio* cardiothoracic ratio, *IVC* inferior vena cava, *LIPV* left inferior pulmonary vein, *Qp/Qs* pulmonary to systemic blood flow ratio, *RPV* right pulmonary veins, *sPAP/sSAP* ratio of main pulmonary arterial systolic pressure to systemic arterial systolic pressure, *TI* time interval between operations

### Preoperative diagnostic studies

All patients were diagnosed with TAPVC using Doppler echocardiography before the first operation. Computed tomography (CT) was performed if any of the 4 pulmonary veins could not be visualized. Patients were monitored via echocardiography for volume overload until the second operation. The second operation was planned if the echocardiography showed a serial increase in cardiac volume or if the patients presented symptoms of heart failure, since the activity of the patients would increase over time. As an exception, a second operation was planned in patients who showed ostial narrowing of the pulmonary vein after the first operation, regardless of volume loading or symptoms (Patient 2 and Patient 4). In 4 patients, additional cardiac catheterization and/or CT was performed just before the second correction for surgical planning (cardiac catheterization alone was performed in 3 patients, while 1 patient underwent both cardiac catheterization and CT). Plain chest radiography was performed in all patients to obtain cardiothoracic ratio data before the second operation.

### Operations

At both stages, all operations were performed under general anesthesia via median sternotomy. Cardiopulmonary bypass was commenced with ascending aortic perfusion and bicaval cannulation. Moderate or deep hypothermia was induced. Cold blood cardioplegia was used for myocardial protection. In all patients, the LSPV, which drained into an innominate vein via a vertical vein, was left uncorrected, and confluent venous drainage from the rest of the anomalous pulmonary veins was directed into the LA during the first operation. The surgical correction for abnormal major drainage to the coronary sinus involved complete unroofing of the coronary sinus wall into the LA and patch closure of the atrial septal defect. In the 2 patients with abnormal major drainage to the inferior vena cava, the vertical vein was ligated and incised to the diaphragm, and then the common pulmonary venous chamber was anastomosed to the rear of the LA. During the second operation, the isolated uncorrected LSPV was anastomosed into the LA auricle, with division of the vertical vein.

## Results

### Preoperative conditions for both operations

At the time of the first operation, the median patient weight was 4.3 kg (range, 3.5–5.4 kg) and the median age was 40 days (range, 20–103 days). The median LSPV size was 4.5 mm (range, 3.0–5.4 mm).

At the time of the second operation, the median patient weight was 12.2 kg (range, 8.5–31.5 kg) and the median age was 1,165 days (range, 280–3,250 days). The median LSPV size was 9.8 mm (range, 8.0–12.3 mm). The median time interval between the operations was 1,094 days (range, 196–3,226 days). The median Qp/Qs in 4 patients who underwent cardiac catheterization just before the second operation was 1.61 (range, 1.22–1.65). The median main pulmonary arterial pressure to systemic arterial pressure ratio was 0.28 (range, 0.24–0.41). The median cardiothoracic ratio was 0.52 (range, 0.49–0.57) on chest radiography before the second operation (Table 1).

### Clinical outcomes and follow-up

The median follow-up period after completion of total correction was 22 months (range, 7–59 months). No postoperative mortality occurred as a result of either operation. One patent (Patient 1) experienced postoperative wound dehiscence after the first operation and underwent delayed sternal wound closure with vacuum-assisted closure. One patient (Patient 6) required prolonged ventilator support for postoperative pulmonary hypertension after the first operation but was discharged after full recovery and underwent a successful second operation. In 2 patients (Patient 2 and Patient 4), the ostia of the pulmonary veins narrowed after the first operation and were relieved using a sutureless technique during the second operation. None of the pulmonary vein anastomosis sites were obstructed after the second operation. On follow-up Doppler echocardiography within 1 week of the second operation, the median V_max_ value at the LSPV anastomosis site was 1.0 m/s (range, 0.8–1.3 m/s), with pulsed flow. Neither turbulent nor continuous flow was observed in any of the patients. The measured median V_max_ value at the same site at the last follow-up Doppler echocardiography was identical to the previous value (1.0 m/s [range, 0.8–1.3 m/s]) (Table 2). The cardiac function was NYHA functional class 1 in all 7 patients during the last follow-up at the outpatient clinic.

**Table 2:** Postoperative outcomes

Patient	Postoperative morbidities		V_max_ at LSPV after 2^nd^ operation (m/sec)		F/U duration after 2^nd^ operation (month)
	1^st^ operation	2^nd^ operation	Before discharge	Last F/U	
1	Wound dehiscence	None	1.3	1.1	59
2	PVO	None	0.8	0.8	18
3	None	None	0.8	0.8	22
4	PVO	None	1.3	1.0	26
5	None	None	1.0	0.9	28
6	PHTN	None	1.1	1.0	7
7	None	None	0.9	1.3	9
Median	N/A	N/A	1.0	1.0	22

*F/U* follow up, *PVO* pulmonary venous obstruction, *PHTN* pulmonary hypertension, *V*_*max*_ maximum velocity on Doppler echocardiography

## Discussion

TAPVC can be classified into 4 subtypes, depending on the drainage site from the pulmonary vein to systemic circulation: supracardiac, infracardiac, cardiac, and mixed type, which are also known as type I to type IV, respectively [16]. Although advances in surgical techniques have improved the surgical outcomes of TAPVC, the optimal surgical management for type IV TAPVC remains controversial [4, 10–12, 17]; the research on this type is challenging because of its rarity, accounting for only 5–13% of all TAPVCs [3–8]. Although type IV TAPVC itself is associated with high mortality risk and/or postoperative pulmonary vein obstruction, the surgical outcomes might also be related to other factors such as patient age, body weight, and the size of the pulmonary veins at the time of surgery [8, 11, 17–21].

St. Louis et al. reported the mortality rate after surgical repair of TAPVC during 3 different time periods between 1982 and 2007, using a multi-institutional database from the Pediatric Cardiac Care Consortium. In their study, the mortality rate decreased as time passed, from 20% between 1982 and 1989 to 16% between 1990 and 1999, and 8% between 2000 and 2007. However, the mortality rate among neonates was still high, with 11.7% mortality in patients younger than 30 days, while the mortality rate after complete correction in patients older than 30 days was 1.7% [5]. Further, Yong et al. reported a consistently high mortality rate after TAPVC repair in neonates during 3 eras (1973 to 1988, 1989 to 1998, and 1999 to 2008) [22]. Surgical repair of TAPVC in neonates has remained a challenge because of the small size of the pulmonary veins.

Surgical repair is inevitable in neonates with TAPVCs such as the obstructive type who require emergent intervention. However, handling of isolated LSPV, which is common in type IV TAPVC, once the other common confluent chamber has been surgically corrected, remains controversial. Some authors reported good results after type IV TAPVC surgery in which the majority of the pulmonary veins were corrected, leaving an isolated small anomalous pulmonary vein uncorrected [4, 12]. However, because left to right shunting from the remaining uncorrected pulmonary vein may increase the risk of heart failure in the long run, the uncorrected isolated anomalous pulmonary vein should eventually be corrected.

Correction of the pulmonary venous confluent chamber buys time for the growth of the LSPV, reducing the risks associated with low body weight, young age, and small size of the LSPV by the time of the second procedure. In the present study, the median size of the LSPV during the second operation was 9.8 mm, which was nearly twice the median size of the LSPV at the first operation (4.6 mm) and close to the size of the LSPV at the ostium in adults, which is 9.6–10.5 mm [23]. Two patients required reoperation for pulmonary venous narrowing at the time of the second operation, which was a complication of the first operation, and not a consequence of leaving the LSPV uncorrected. The median V_max_ of the anastomosed LSPV to the LA auricle within 1 week after the second operation was 1.0 m/s, without any turbulent or continuous flow, indicating that there was no narrowing or obstruction [24, 25]. This patency was maintained at the last follow-up, showing an identical median V_max_ of 1.0 m/s (Table 2).

Considering that the median Qp/Qs was 1.6 owing to the left to right shunt from the uncorrected LSPV, correction of the LSPV is recommended. Until the LSPV grows to an appropriate size for correction, patients need to be monitored closely to avoid disease progression, leading to heart failure. We had planned to perform the second operation when volume overload would be suspected, and also when the patients would complain of symptoms, as their activity would increase with time. Interestingly, in the present study, all second operations were planned in patients without any symptoms; the patients were monitored through echocardiography, CT, and angiography, including 2 patients (Patient 2 and Patient 4) who had the ostium of the pulmonary veins narrowed after the first operation. This is another evidence of the importance of close monitoring.

### Limitations

The present study is a retrospective and descriptive study with a small sample size. Owing to the low incidence of type IV TAPVC, obtaining a large sample with type IV TAPVC is difficult. Hence, the dates of operation were spread out across a long period of time. Late follow-up was not completed in recent cases. As a result, there was a huge variability in the follow-up period. Nevertheless, this study describes successful short-term and mid-term results of two-stage surgical strategy for type IV TAPVC, without any major complications. The mortality rate after surgical correction in type IV TAPVC is reported to be 8–42% [9–11]; however, the surgical strategy used in these studies was unclear. To the best of our knowledge, only one previous case of two-stage correction of type IV TAPVC has been reported [26]. In this context, although this is a small series, it confirms the practice of delaying complete repair in order to mitigate the problematic complication of pulmonary venous obstruction, which is frequently observed in patients who undergo repair of TAPVC during the neonatal period.

## Conclusions

We performed two-stage correction of type IV TAPVC, with satisfactory results. Leaving an isolated anomalous pulmonary vein uncorrected during the first operation and performing a second operation when this pulmonary vein has grown adequately for anastomosis could be a useful strategy for surgical repair of type IV TAPVC.

## Abbreviations

CT: Computed tomography
LA: Left atrium
LSPV: Left superior pulmonary vein
PVO: Pulmonary vein obstruction
TAPVC: Total anomalous pulmonary venous connection
